# Loop-mediated isothermal amplification combined with lateral flow biosensor for rapid and sensitive detection of monkeypox virus

**DOI:** 10.3389/fpubh.2023.1132896

**Published:** 2023-03-24

**Authors:** Xiaolan Huang, Fei Xiao, Nan Jia, Chunrong Sun, Jin Fu, Zheng Xu, Xiaodai Cui, Hui Huang, Dong Qu, Juan Zhou, Yi Wang

**Affiliations:** ^1^Experimental Research Center, Capital Institute of Pediatrics, Beijing, China; ^2^Department of Infectious Diseases, Children’s Hospital Affiliated Capital Institute of Pediatrics, Beijing, China; ^3^Department of Critical Medicine, Children’s Hospital Affiliated Capital Institute of Pediatrics, Beijing, China

**Keywords:** monkeypox virus, loop-mediated isothermal amplification, lateral flow biosensor, nanoparticles, diagnosis

## Abstract

The ongoing outbreak of the monkeypox, caused by monkeypox virus (MPXV), has been a public health emergency of international concern, indicating an urgent need for rapid and sensitive MPXV detection. Here, we designed a diagnostic test based on loop-mediated isothermal amplification (LAMP) and nanoparticle-based lateral flow biosensor(LFB)for diagnosis of MPXV infection, termed MPX-LAMP-LFB. A set of six LAMP primers was designed based the ATI gene of MPXV, and LAMP amplification of MPXV templates was performed at 63°C for only 40 min. The results were rapidly and visually decided using the LFB test within 2 min. The MPX-LAMP-LFB assay can specifically detect MPXV strains without cross-reaction with non-MPXV pathogens. The sensitivity of the MPX-LAMP-LFB assay is as low as 5 copies/μl of plasmid template and 12.5 copies/μl of pseudovirus in human blood samples. The whole process of the MPX-LAMP-LFB assay could be completed ~1 h, including rapid template preparation (15 min), LAMP reaction (40 min)and result reporting (<2 min). Collectively, MPX-LAMP-LFB assay developed here is a useful tool for rapid and reliable diagnosis of MPXV infection.

## Introduction

The monkeypox virus (MPXV), which belongs to the family *Poxviridae*, subfamily *Chordopoxvirinae*, and genus *Orthopoxvirus*, causes monkeypox (MPOX) (1). MPXV was first isolated from cynomolgus monkeys in 1958 and the first human infection case was reported in 1970 (2). MPOX presented symptoms similar to those of smallpox in humans (3). People infected usually started with fever, myalgia, fatigue and headache, followed by macular papules. MPXV infection was typically endemic to the rainforests of Central and West Africa, where a number of ground squirrel species believed to be the hosts are prevalent (4). More recently, MPOX outbreak has occurred in 110 Member States across all 6 WHO (World Health Organization) regions, including 103 non-endemic countries/regions, as of December 23, a total of 83,497 laboratory confirmed cases (including 72 deaths) has been reported^1^. Particularly, MPXV has been declared a global health emergency by the WHO, in the context of the COVID-19 pandemic (5, 6). Therefore, it is important to develop a simple and efficient laboratory detection method for MPXV detection.

As a re-emerging virus, a number of technologies have been developed for MPXV detection, including viral culture and isolation, electron microscopy, immunohistochemistry and enzyme linked immunosorbent assay (ELISA) and molecular tests. However, these assays are all time-consuming and needed to be performed at a central laboratory with skilled technicians and sophisticated instruments (7). Thus, further establishment of easy-to-use, more rapid and simpler technologies to diagnose MPXV infection are still required for facilitating infection control, clinical care and epidemiologic investigation.

Loop mediated isothermal amplification (LAMP), as one of the most commonly used isothermal detection methods for various pathogens, allows amplification of templates, including DNA and RNA, with high sensitivity and specificity at a fixed temperature of 60–69°C. LAMP results can be reported by the use of spectrophotometric equipment for measuring turbidity, by agarose gel electrophoresis, or by visual inspection of color on turbidity changes (8). However, gel electrophoresis is time consuming, laborious and equipment demanding. Visual inspection with colorimetric indicators were direct and convenient, but visual inspection is subjective and sometimes it is difficult to decide the results through an inconspicuous color change. The spectrophotometric turbidimeters can real-time monitor the turbidity changes and yield quantitative results, but the apparatuses are expensive which limited its popularity.

To help overcome these problems caused by traditional LAMP monitoring methods, nanoparticle-based lateral flow biosensor (LFB) was successfully designed and applied for reporting LAMP results. LFB is a paper-based biosensor, which has the advantages of good robustness, fast speed, cost-effectiveness, high specificity and sensitivity (9, 10). Moreover, reporting LAMP results using LFB permits the visual readout, eliminating the use of sophisticated apparatus (11). Therefore, in this study, a LAMP amplification coupled with a LFB assay was established for the rapid and simple detection of MPXV, termed MPX-LAMP-LFB. The features of simplicity (without the requirement for expensive or complex apparatus), rapidity (results can be reported within 60 min), and excellent specificity and sensitivity make this MPX-LAMP-LFB assay more suitable for application in low-equipment setting laboratory.

## Materials and methods

### Reagents and instruments

A standard plasmid (ATI-plasmid), containing partial sequence of ATI gene(GenBank accession: MT903346.1) shared by both the Clade one (I) (the former Congo Basin (Central African) clade) and the Clade two (II) (the former West African clade) of the causative agent of MPOX, was constructed by Tianyi-Huiyuan Biotech Co., Ltd. (Beijing, China). A pseudotyped virus, which also contained the ATI gene, was constructed by Sangon Biotech. Co., Ltd. (Shanghai, China). Both common and labeled primers used in this study were synthesized by AOKE Biotech Co., Ltd. (Beijing, China). Visual indicator (VI) [used as visual detection regent (VDR)], DNA Isothermal Amplification Kit and LFB were all provided by HUIDEXIN Biotech Co., Ltd. (Tianjin, China). Genomic DNA kit for nucleic acid extraction and purification was purchased from Beijing TransGen Biotech Co., Ltd. (Beijing, China). Real-time turbidimeter LA-320C was purchased from Eiken Chemical Co., Ltd., Japan.

### Primer design

**Figure 1:**
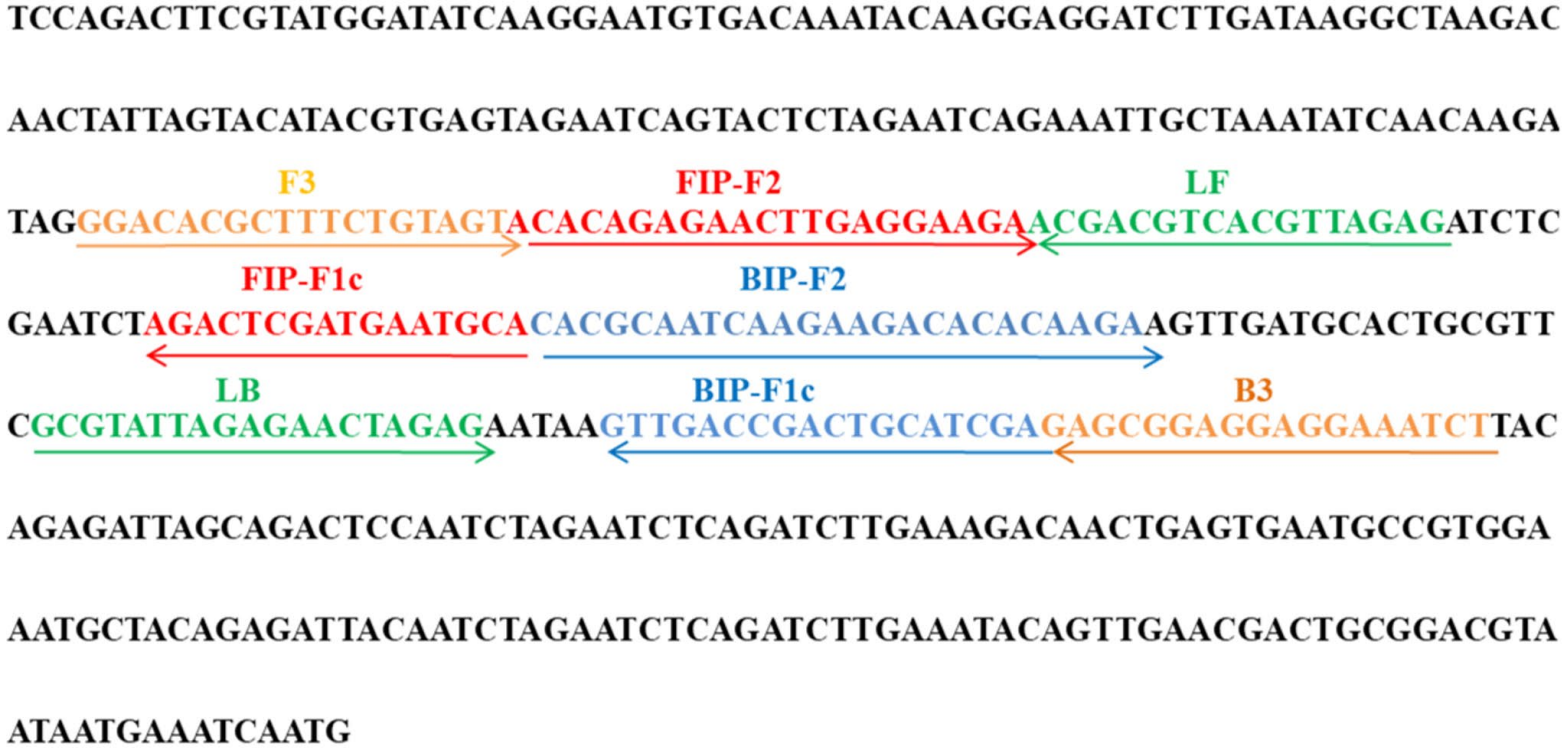
Primer sequences and locations used in this study. Right and left arrows show sense and complementary sequences, respectively. The colored text indicates the position of primers, including two outer primers (F3 and B3), two inner primers (FIP and BIP) and two loop primers(LF and LB).

A set of 6 primers, including two outer primers (F3 and B3), two inner primers (FIP/FIP* and BIP) and two loop primers (LF/LF* and LB), were designed based on the ATI gene of MPXV using Primer Premier 5.0 (12) for LAMP reaction, and a set of primers for conventional PCR was also designed using the Primer-Blast tool of NCBI for comparison. All the designed primer sets were subjected to blast against the NCBI database, the primer sets that nonspecifically matched with other microorganisms were excluded and the optimal ones were achieved. The sequences, locations and modifications of the primers used in this report are shown in Figure 1 and Table 1.

**Table 1:** Sequences and modifications of the MPX-LAMP-LFB primers.

Primers^a^	Sequence (5′-3′) and modifications	Length^b^
F3	GGACACGCTTTCTGTAGT	18 nt
B3	AGATTTCCTCCTCCGCTC	18 nt
FIP	TGCATTCATCGAGTCTAGATTCGACACAGAGAACTTGAGGAAGA	44 mer
FIP*	FAM-TGCATTCATCGAGTCTAGATTCGACACAGAGAACTTGAGGAAGA	46 mer
BIP	CACGCAATCAAGAAGACACACAAGATCGATGCAGTCGGTCAAC	43 mer
LF	CTCTAACGTGACGTCGT	17 nt
LF*	Biotin-CTCTAACGTGACGTCGT	21 nt
LB	GCGTATTAGAGAACTAGAG	19 nt
F	ATCAACAAGATAGGGACACG	20 nt
R	TGCAGTCGGTCAACTTATTC	20 nt

^a^F3, forward outer primer; FIP, forward inner primer; LF, forward loop primer; LB, backward loop primer; BIP, backward inner primer; B3, backward outer primer; FIP*, 5′-labeled with carboxyfluorescein when used in the COM-MPXV-LAMP-LFB assay; LF*, 5′-labeled with biotin when used in COM-MPXV-LAMP-LFB assay. F and R, the forward and reverse primers used for conventional PCR. ^b^nt, nucleotide; mer, monomeric unit.

### Nucleic acid extraction

According to the manufacturers’ instructions, the nucleic acid templates were extracted from blood samples using the EasyPure^®^Viral DNA/ RNA Kit purchased from Beijing TransGen Biotech Co., Ltd. (Beijing, China) and from swab samples using nucleic acid extraction reagent purchased from Capital Bio Technology Co., Ltd. (Sichuan, China). The extracted templates were stored under at −20°C before use.

### Preparation of lateral flow biosensor

The nanoparticle-based lateral flow biosensor (LFB) was prepared as previously described (13–15). Briefly, the four components of LFB, including a sample pad, a conjugate pad, a nitrocellulose membrane and an absorbent pad (Jie-Yi Biotechnology), were sequentially assembled on plastic back card. The conjugated region contained dye streptavidin coated polymer nanoparticles (SA-DNPs, 129 nm, 10 mg mL^-1^, 100 mM borate, pH 8.5 with 0.1% BSA, 0.05% Tween 20 and 10 mM EDTA; Bangs, Laboratories, Inc. Indiana, United States), which was employed as detector regents for visualization of targets. The nitrocellulose membrane (NC), which functioned as the detection region, contained rabbit anti-carboxyfluorescein antibody (anti-FAM, 0.2 mg/ml, Abcam. Co. Ltd.) at the test line (TL) and biotinylated bovine serum albumin (biotin-BSA, 4 mg/ml, Abcam. Co. Ltd.) at the control line (CL), with a distance of 5 mm between the two lines. The assembled LFB were cut into 4-mm dipsticks, packaged in a plastic cassette and stored at room temperature until use.

For reporting the amplicons, an aliquot of 5 μl amplification products was added to the sample region of LFB, followed by a 100 μl aliquot of running buffer (10 mM PBS, PH 7.4 with 1% Tween 20) to the same region. Under the capillary force, the target amplicons, which were labelled with FAM and biotin at each end, were specially captured by the anti-FAM of the NC region and visualized through biotin and SA-DNPs interaction, resulting to a red band at TL region. The remained SA-DNPs was captured at CL region, indicating the availability of LFB.

### The standard MPX-LAMP-LFB reaction

The plasmid DNA was used as positive control to establish the standard MPX-LAMP-LFB assay. The LAMP assay was performed in a 25 μl reaction mixture containing 12.5 μl 2 × reaction buffer, 0.1 μM (each) of F3 and B3 primers, 0.4 μM (each) of FIP* and BIP primers, 0.2 μM (each) of LF* and LB primers, 1.0 μl *Bst* DNA polymerase (8 U), 1.2 μl VDR, 1.0 μl template (5.0 μl in sample detection) and 7.3 μl distilled water (DW). The mixtures were incubated at 63°C for 1 h, and then at 80°C for 5 min to terminate the reaction. Mixture with 1.0 μL genomic DNA of influenza virus A was used as negative control, and with 1 μl DW as the blank control.

The LAMP results were monitored by real-time turbidity, visual reagent (VDR) and LFB. In brief, LFB contains a sample pad, a conjugated pad, a nitrocellulose membrane and an absorbent pad (10, 13). When the reaction products (5 μl) were added in the sample pad, following with about 100 μl running buffers, the results were indicated with two red lines (CL and TL) representing positive reactions, or negative with only one line at CL.

### Optimal temperature of MPX-LAMP-LFB assay

Temperatures ranging from 60 to 67°C (with an interval of 1°C) were examined to determine the optimal temperature using the standard LAMP reaction system. LAMP reactions were conducted using the real-time turbidimeter LA-320, with a threshold value of >0.1 as positive reaction. A mixture with DW was regarded as blank control. Each test was repeated three times.

### Specificity of the MPX-LAMP-LFB assay

A total of 19 DNA templates, including the ATI-plasmid, pseudotyped virus of MPXV and 17 other virus (Supplementary Table S1) were used to evaluate the analytical specificity of the MPX-LAMP-LFB assay. Genomic DNA of the entire virus was extracted according to the manufactures’ instruction. Each test was performed in triplicate.

### Sensitivity of the MPX-LAMP-LFB assay

The ATI-plasmid and the pseudotyped virus were serially 10-fold diluted (5 × 10^5^ ~ 5 × 10^−1^copiesper microliter for ATI-plasmid and 1.25 × 10^3^ ~ 1.25 × 10^−4^copiesper microliter for the pseudotyped virus) to determine the detection limit of the MPX-LAMP assay. Each serial dilution was tested in duplicate to verify the limit level of the MPX-LAMP-LFB assay. Results of the LAMP reactions were reported by LBF, and further confirmed by real-time turbidity and VDR test. Each test was repeated three times.

### Optimal amplification time of MPXV-LAMP–LFB assay

The serially diluted templates of the ATI-plasmid were examined at 63°C to optimize the amplification time of MPX-LAMP-LFB assay. Reaction products were continually analyzed using VDR and LFB test at the 10–40 min with an interval of 10 min. Each reaction was tested in triplicate.

### Application of the MPX-LAMP-LFB assay in simulated specimens

In order to verify the feasibility of the MPX-LAMP-LFB assay for clinical diagnosis of MPVX infection, the previously diluted pseudo typed viruses were incubated into human blood samples and lesional swab samples preparing the simulated specimens. Then, the tainted blood and swab samples were applied for nucleic acid extractions, and the resultant supernatants were used as templates for the MPX-LAMP-LFB assay, with the unsimulated samples as blank controls. Moreover, the direct simulated blood and swab samples were further detected by the MPX-LAMP-LFB assay without nucleic acid extraction to test the potential of point-of-care usability of this assay. In addition, 61 nasopharyngeal swab (NPS) samples of non-MPXV infection were enrolled in the process for the evaluation of the MPX-LAMP-LFB assay in the clinical setting. The clinical samples were collected from children in the clinics of the Children’s Hospital of Capital Institute of Pediatrics from 1 February to 30 December in 2022, the ethical practice was approved by the Ethical Committee of Capital Institute of Pediatrics, and all the samples were obtained within formed consents signed by the participants’ guardians.

### Conventional PCR

For confirmation and comparison, a novel conventional PCR method targeting the same gene region of the MPX-LAMP-LFB assay was also developed and used to test the standard ATI-plasmid and the pseudotyped viruses. The conventional PCR reaction was performed in a reaction mixture of 25 μl containing 12.5 μl Premix Ex Taq™ II (Takara Bio, Inc., Otsu, Japan), 0.4 μM F primer, 0.4 μM R primer and 1 μl of DNA template. The conventional PCR reaction procedure contained pre-denaturation at 95°C for 30 s, 30 cycles of denaturation at 95°C for 30 s, annealing at 58°C for 30 s and extension at 72°C for 30 s, and a final extension at 72°C for 5 min. Then, 5 μl of the reaction products were separated by 2% agarose gel electrophoresis (120 V, 40 min). Images were taken using a gel imaging system (GelDoc TM XR1 imager, Bio-Rad Laboratories, Co., Ltd.). The positive result should show a visible band of 177 bp. For precise confirmation of the products, the reaction products were sequenced by Sanger sequencing method provided by Tianyi-Huiyuan Biotech Co., Ltd. (Beijing, China) and the returned sequences were subjected to sequence similarity searching using the Blast tool of NCBI website. In addition to for analytical sensitivity comparison, the conventional PCR was applied to confirm the clinical NPS samples non-MPXV infected.

## Results

### Confirmation of effectiveness of the MPX-LAMP-LFB assay

**Figure 2:**
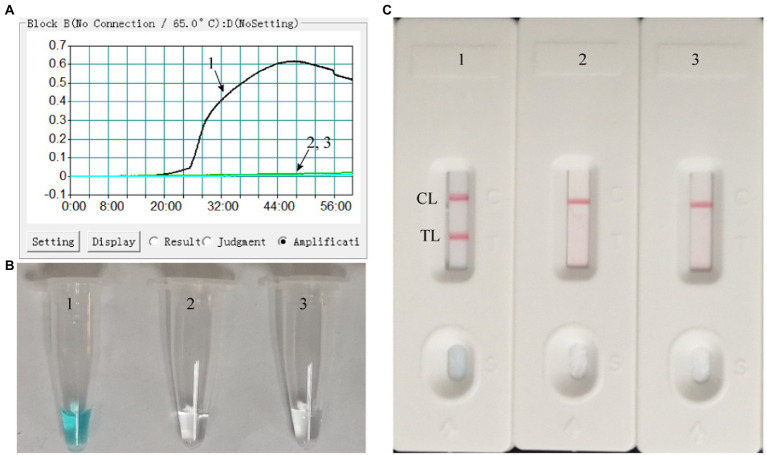
Effectiveness of the primer set for the MPX-LAMP-LFB assay. The effectiveness of the primer set for the MPX-LAMP-LFB assay was verified by testing the LAMP products with real-time turbidity **(A)**, visual detection reagent **(B)**, and LFB **(C)** methods. Curve/Tube/Biosensor 1, the ATI-plasmid that used as positive control, which was effectively amplified with LAMP reaction at 65°C; Curve/Tube/Biosensor2, the genomic DNA of influenza virus A that used as negative control; Curve/Tube/Biosensor3, the blank control (DW).

The LAMP reaction was performed at 65°C for 60 min to validate the feasibility of the designed primers. Using real-time turbidimeter, a significant increase of turbidity was observed in the mixture with templates of ATI-plasmid, while an almost blunt curve was seen in the negative and blank control (Figure 2A). Using VDR, the color shift of positive results in LAMP tubes from colorless to light green was observed with naked eyes after incubation, and negative results in LAMP tubes remained colorless (Figure 2B). Using LFB, two red bands (CL and TL) were visible in positive results, but only CL was seen in negative and blank controls (Figure 2C). These data above suggested that the selected primers were effective-sufficient for detection of MPXV using LAMP-based assay. In addition, by using the primers of conventional PCR, a visible band was only present in the mixture with templates of ATI-plasmid (Supplementary Figure S1). After sequencing and alignment, it was confirmed that the products was particle region of the ATI-plasmid, implying the availability of the conventional PCR for MPXV test.

### Optimal temperature of the MPX-LAMP-LFB assay

We performed the MPX-LAMP-LFB assay at eight different temperatures ranging from 60 to 67°C with a 1°C interval for the optimization of the reaction temperature. As shown in Supplementary Figure S2, 63°C was concluded to be the optimal temperature for the MPX-LAMP-LFB assay, since the threshold value of 0.1 of absorbance was achieved fastest at this condition. Therefore, 63°C was used for the subsequent MPX-LAMP-LFB reaction conducted in this report.

### Specificity of MPX-LAMP-LFB assay

**Figure 3:**
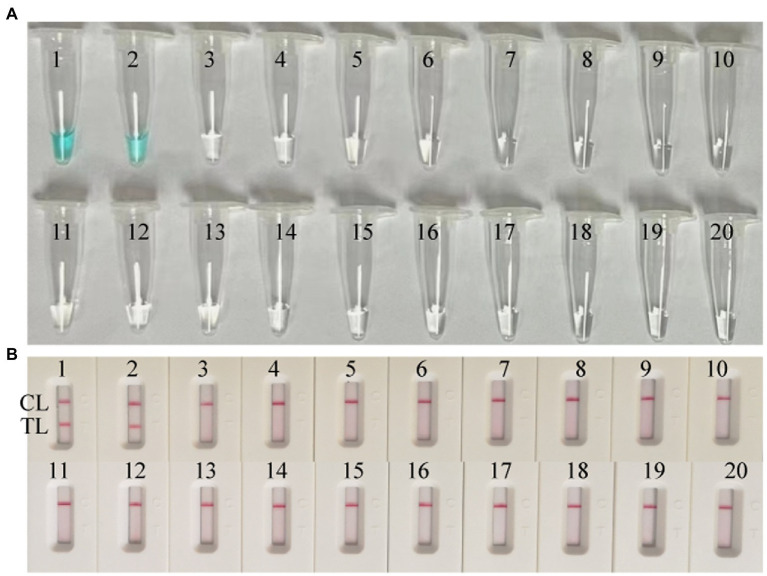
Specificity conformation for MPX–LAMP-LFB assay. Specificity of the MPX-LAMP-LFB assay was confirmed by testing the LAMP products with visual detection reagent **(A)** and LFB **(B)** methods. Biosensors/Tubes 1–2 showed the LAMP reaction results of ATI-plasmid and the pseudotyped virus; Biosensors/Tubes 3–19 showed the LAMP reaction results of the 17 non-MPXV viral pathogens (Supplementary Table S1); Biosensor/Tube 20 showed the LAMP reaction results of blank control. TL, test line; CL, control line.

DNA templates of MPXV (ATI-plasmid and the pseudotyped virus)and non-MPXV viruses were used to estimate the specificity of the MPX-LAMP-LFB assay under the optimal conditions confirmed above. The results were confirmed using LFB test. Only reactions using templates of ATI-plasmid and pseudotyped virus demonstrated positive results (Figure 3). No cross-reaction was observed within the non-MPXV viruses (Supplementary Table S1).

### Sensitivity of the MPX-LAMP-LFB assay

**Figure 4:**
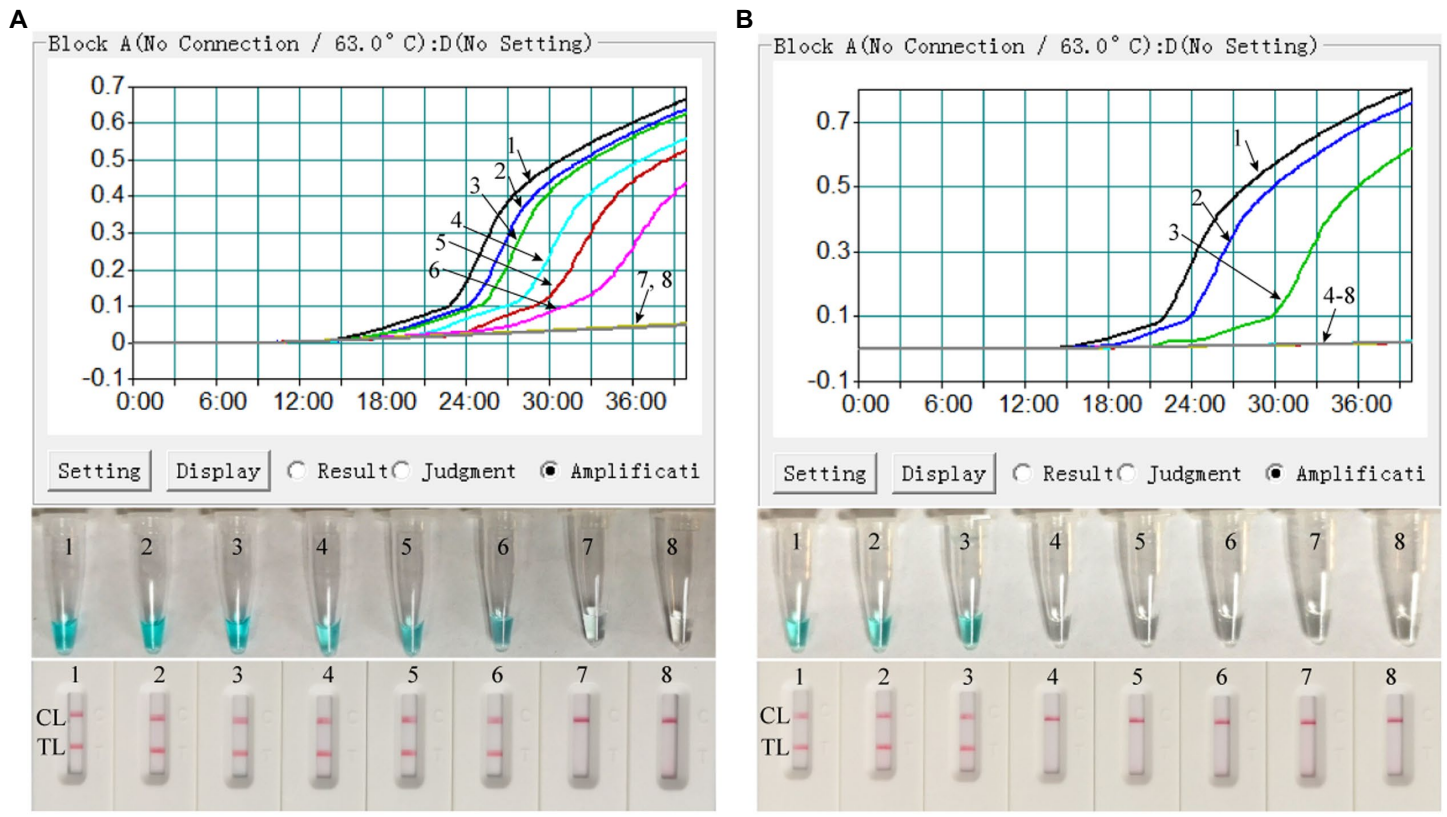
Sensitivity confirmation of MPX–LAMP-LFB assay. The sensitivity of the MPX–LAMP-LFB assay was assessed by using the serially diluted ATI-plasmid **(A)** and the constructed pseudotyped virus **(B)**. Three monitoring formats, including turbidity (up row), visual detection reagent (middle row), and LFB (bottom row), were applied to detect LAMP products. Templates 1–8 in A were the ATI-plasmid with concentrations of 5 × 10^5^, 5 × 10^4^, 5 × 10^3^, 5 × 10^2^, 5 × 10^1^, 5 × 10^0^, and 5 × 10^−1^copies permicroliter and the uncontaminated blood sample; Templates 1–8 in B were the constructed pseudotyped virus with concentrations of 1.25 × 10^3^, 1.25 × 10^2^, 1.25 × 10^1^, 1.25 × 10^0^, 1.25 × 10^−1^, 1.25 × 10^−2^, and 1.25 × 10^−3^copies per microliter and the uncontaminated lesional swab sample. TL, test line; CL, control line.

Both the ATI-plasmid and the pseudotyped virus were serially diluted for the examination of the detection limit of the assay. As shown in Figure 4, the results detected by the LFB showed that the detection limit of the MPX-LAMP-LFB assay was 5 × 10^0^copies/μl and that of the pseudotyped virus was 1.25 × 10^1^ copies/μl per reaction, in accordance with those indicated by turbidity and VDR and lower than that of conventional PCR (5 × 10^1^ copies/μl of plasmid template and that of the was 1.25 × 10^2^ copies/μl pseudotyped virus template, Supplementary Figure S3).

### Optimal time of the MPX-LAMP-LFB assay

As shown in Supplementary Figure S4, at the time of 40 min, the products of the mixtures with the detection limit level of template were successfully detected by the VDR and LFB test. Thus, 40 min was selected as the optimal incubation time for the MPX-LAMP reaction. Consequently, the whole procedure of MPX-LAMP-LFB analysis only needs no more than 60 min, including rapid DNA extraction (15 min), isothermal reaction (40 min), and result indication (2 min).

### Application of MPX-LAMP-LFB assay in simulated specimens

**Figure 5:**
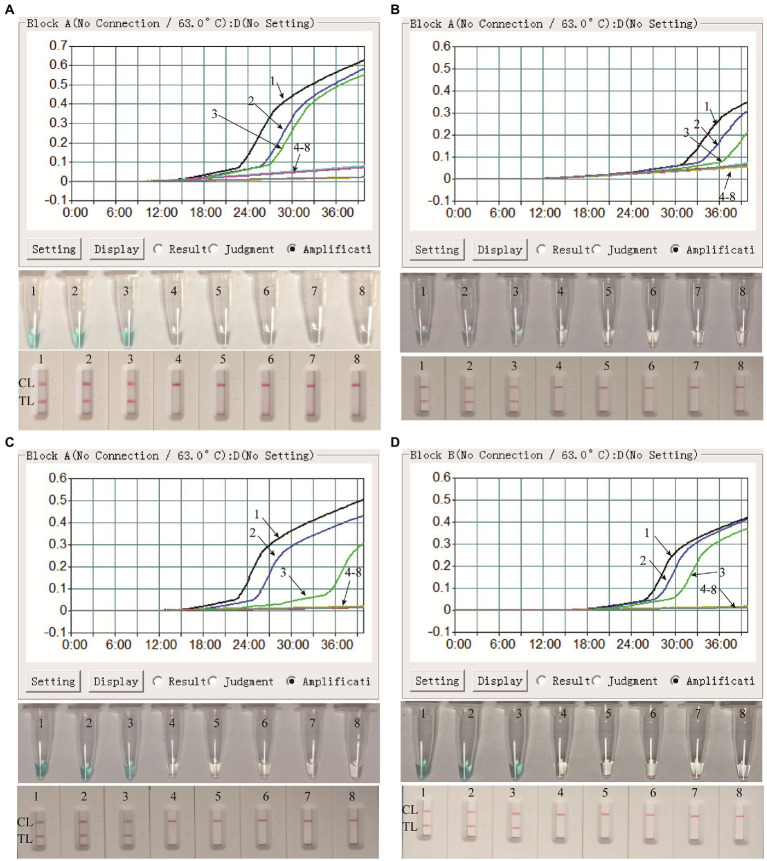
Clinical feasibility confirmation of the MPX-LAMP-LFB assay in simulated blood and lesional swab specimens. Clinical feasibility of the MPX-LAMP-LFB assay was confirmed by using the serially diluted simulated blood specimens with nucleic acid extraction process **(A)** and without nucleic acid extraction process **(B)**, and the serially diluted simulated lesional swab specimens with nucleic acid extraction process **(C)** and without nucleic acid extraction process **(D)**. All the specimens were detected using turbidity (top row), visual detection reagent (middle row)and LFB (bottom row). Curves/Tubes/Biosensors 1–8 represented the amplification results of the pseudotyped virus levels in blood samples of 1.25 × 10^3^, 1.25 × 10^2^, 1.25 × 10^1^, 1.25 × 10^0^, 1.25 × 10^−1^, 1.25 × 10^−2^, and 1.25 × 10^−3^copies permicroliter and DW.TL, test line; CL, control line.

In order to confirm the availability of clinical application, the optimized MPX-LAMP-LFB assay were applied to test the artificial tainted blood and lesional swab samples and 61 clinical NPS samples. The results showed that the MPX-LAMP-LFB assay can effectively detect the pseudotyped virus in the simulated blood and lesional swab samples before or after nucleic acid extraction with the detection limit of 1.25 × 10^1^ copies, which was identical to that of the pure pseudotyped MPXV (Figure 5). Of note, although results from detection of the direct blood samples by real-time turbidity and VDR methods was somewhat weak positive and difficult to recognize, the ones by LFB method were unambiguous and easy to read. Moreover, all the clinical samples diagnosed as non-MPXV infection by conventional PCR (Supplementary Figure S5) were also detected negative of MPXV by MPX-LAMP-LFB (Supplementary Figure S6), indicating the high specificity of the MPX-LAMP-LFB assay.

## Discussion

Since the beginning of May 2022, human MPOX outbreaks reemerged and have spread to many countries, even as to areas where the disease was not endemic previously (16). The reemergence of the human MPOX outbreak has made the MPXV become the focus of attention again. Timely and accurate identification of those infected with MPXV is critical to prevent further spread of the disease. Here, we developed a rapid, ultra-sensitive, specific and user-friendly method for the diagnosis of MPXV and named it the MPX-LAMP-LFB assay.

In the MPX-LAMP-LFB assay, isothermal LAMP technique was used to amplify target sequences and LFB was used for indicating the results. In order to perform the MPX-LAMP-LFB test, only a simple instrument (such as a hot block or water bath) is required, maintaining a constant temperature (63°C). Particularly, LAMP assay can achieve exponential amplification of target template within 40 min (8, 17, 18). What’s more, the nanoparticle-based LFB platform could visually and objectively decode the pre-amplified targets without expensive and professional laboratory conditions (9). The feature of not limited by site and instrument enables the LFB test to avoid workplace and equipment contamination, which always presents through transfer of nucleic acid amplification test products. In our study, we carried out the LFB test in the clean bench apart from the amplification room, which effectively avoid contamination from the products. The used LFB strips could be easily handled without contamination of any equipment. The whole detection process, including rapid sample processing (15 min), LAMP reaction (40 min), and LFB detection (2 min), could be completed within 60 min. Therefore, the MPX-LAMP-LFB test was a simple, rapid and user-friendly method and may be applied in resource-limited areas or bed-site for MPX infections diagnosis.

Sensitivity analysis showed that the MPX-LAMP-LFB assay had high sensitivity in detecting MPXV. The MPX-LAMP-LFB assay could detect as low as 5 copies of pure plasmid templates and 12.5 copies of pure pseudotyped virus templates. Obviously, the MPX-LAMP-LFB method was highly sensitive for MPXV detection, which was comparable to the real-time PCR based method (∼3.5 genomes) (19), and more sensitive than the newly developed conventional PCR method and the previously reported LAMP-based method (10^2^ ~ 10^3^copies per reaction) (20) and RPA-based method (16 molecules per reaction) (21). In addition, the MPX-LAMP-LFB assay correctly reported negative results for the 17 non-MPXV virus, suggesting the highly specificity for MPXV detection. Moreover, although no available non-MPXV *Orthopoxvirus* was obtained, the specificity of the MPX-LAMP-LFB assay between MPXV and other *Orthopoxvirus* members was confirmed by using the blast tool of NCBI database to obtain sequence similarity between the amplified sequence of MPXV strains and that of the other *Orthopoxvirus* members (Supplementary Table S2 and Supplementary Figure S7). It will be preferable if non-MPXV *Orthopoxvirus* strains were obtained and validated in the future study.

Simulated blood and lesional swab specimens were tested to reveal the feasibility of MPX-LAMP-LFB assay in clinical practice. No matter in the directed simulated samples or the ones with nucleic acid extraction process, the MPX-LAMP-LFB assay can successfully characterize and identify the presence of the pseudovirus of MPXV. Moreover, the MPX-LAMP-LFB assay was able to test positive in the simulated blood and lesional swab specimens with down to 12.5 copies of pseudovirus, which further verifying the high sensitivity of this method in clinical settings. In addition, negative results of 61swabs collected from non-MPXV infected patients further demonstrate the analytical specificity of the MPXV-LAMP-LFB assay. The feasibility of MPX-LAMP-LFB assay in clinical settings enables more opportunity to rapid and accurate identify the individuals with MPOX, especially the application in direct simulated blood and lesional swab specimens, implying its great potential in bed-site settings for MPXV infection diagnosis. Thus, the MPXV carriers can be isolated and treated timely, avoiding cross infection and further spread of MPXV, which is of vital importance for the MPOX surveillance in the world.

In summary, we combined LAMP with LFB to develop a diagnostic method for MPXV infection (termed MPX-LAMP-LFB assay). The results showed that the MPX-LAMP-LFB assay was a rapid, efficient, sensitive, specific and simple method for the detection of MPXV infection, and could be used for the detection of MPXV infection in clinic. The MPX-LAMP-LFB assay does not require complex instruments or skilled technicians and can be completed within 60 min. Therefore, the MPX-LAMP-LFB test developed here is an effective tool for the rapid and reliable diagnosis of MPXV infection in both the scientific research field and clinical settings, especially for point-of-care testing or in rough conditions.

## Data availability statement

The original contributions presented in the study are included in the article/Supplementary material, further inquiries can be directed to the corresponding authors.

## Ethics statement

This study was approved by the Ethical Committee of Capital Institute of Pediatrics, and all the samples were obtained with informed consents signed by the participants’ guardians.

## Author contributions

XH performed the experiments, analyzed the data, and drafted the manuscript. FX and NJ performed the experiments. CS analyzed the data and drafted the manuscript. JF, ZX, and XC contributed reagents and materials. HH, DQ, and JZ supervised the clinical guidance and study, as well as revised the manuscript. YW conceived the study, designed the experiments, revised the manuscript, supervised and funded this study. All authors contributed to the article and approved the submitted version.

## Funding

This study was funded by Beijing Nova Program (Z211100002121042), National Key Research and Development Program of China [Grant Nos. 2021YFC2301101 (YW) and 2021YFC2301102 (YW)].

## Conflict of interest

The authors declare that the research was conducted in the absence of any commercial or financial relationships that could be construed as a potential conflict of interest.

## Publisher’s note

All claims expressed in this article are solely those of the authors and do not necessarily represent those of their affiliated organizations, or those of the publisher, the editors and the reviewers. Any product that may be evaluated in this article, or claim that may be made by its manufacturer, is not guaranteed or endorsed by the publisher.

## Footnotes

^1^ https://worldhealthorg.shinyapps.io/mpx_global/
