# Supplementary material for: Loop-mediated isothermal amplification combined with lateral flow biosensor for rapid and sensitive detection of monkeypox virus
Source: Front Public Health. 2023 Mar 24;11:1132896. doi: 10.3389/fpubh.2023.1132896 (PMC10080115; doi:10.3389/fpubh.2023.1132896)
Supplement: Supplementary file 1 [file Data_Sheet_1.docx]

**Loop-mediated isothermal amplification combined with lateral flow biosensor for rapid and sensitive detection ofMonkeypox virus**

Xiaolan Huang^1^, Fei Xiao^1^, Nan Jia^1^, Chunrong Sun^1^, Jin Fu^1^, Zheng Xu^1^, Xiaodai Cui^1^, Hui Huang^2*^, Dong Qu ^3*^, Juan Zhou^1,*^ and Yi Wang ^1*^

^1^ Experimental research center, Capital Institute of pediatrics, Beijing, 100020, P.R. China

^2^ Department of Infectious Diseases, Affiliated Children’s Hospital, Capital Institute of Pediatrics, Beijing, 10020, P. R. China

^3^Department of Critical Medicine, Children’s Hospital Affiliated Capital Institute of Pediatrics, Beijing 100020, P.R. China.

*Correspondence: Yi Wang, wildwolf0101@163.com (Handing the Correspondence)

Dong Qu, qudong2012@126.com

Hui Huang, [huihui07@tom.com](mailto:huihui07@tom.com)

Juan Zhou, zhoujuan2015@126.com

**Tables**

**Table S1 Strains used for specificity confirmation of the MPX-LAMP-LFB assay**

| **No.** | **Pathogen** | **Strain no. (source of the strains) ^a^** | **MPX-LAMP-LFB^b^** |
| --- | --- | --- | --- |
| 1 | ATI-plasmid | CIP | P |
| 2 | pseudotypedvirus | CIP | P |
| 3 | Parainfluenza3 | Isolated strains (CDC) | N |
| 4 | Hepatitis B virus | Isolated strains (CDC) | N |
| 5 | Measles virus | Isolated strains (CDC) | N |
| 6 | Parainfluenza 1 | Isolated strains (CDC) | N |
| 7 | Influenza B virus | Isolated strains (CDC) | N |
| 8 | Coxsackie virus | Isolated strains (CDC) | N |
| 9 | Rotavirus | Isolated strains (CDC) | N |
| 10 | Epstein-Barr virus | Isolated strains (CDC) | N |
| 11 | Human rhinovirus | Isolated strains (CDC) | N |
| 12 | Herpes simplex virus 1 | Isolated strains (CDC) | N |
| 13 | Respiratory syncytial virus | Isolated strains (CDC) | N |
| 14 | Adenovirus 9 | Isolated strains (CIP) | N |
| 15 | Influenza A virus | Isolated strains (CIP) | N |
| 16 | Murine Respirovirus | Isolated strains (CIP) | N |
| 17 | Visna virus | Isolated strains (CIP) | N |
| 18 | Vesicular stomatitis virus | Isolated strains (CIP) | N |
| 19 | Dengue virus | Isolated strains (CIP) | N |

**^a^**CIP, Capital Institute of Pediatrics; CDC, Chinese Center for Disease Control and prevention.

**^b^**P, positive; N, negative.

**Table S2 Cross-Reactivity/Exclusivity In silico Results**

| Virus | Strain | GenBank | ATI gene similarity |
| --- | --- | --- | --- |
| Monkeypox virus | MPXV-USA2003_099_Gambian_Rat | MT903346.1 | 100% |
| Rabbitpox virus | - | AY484669.1 | 96.20% |
| Camelpox virus | Negev2016 | MK910851.1 | 95.70% |
| Horsepox virus | MNR | KY349117.1 | 95.70% |
| Cowpox virus | HumBer07/1 | KC813509.1 | 95.20% |
| Taterapox virus | - | NC_008291.1 | 95.20% |
| OrthopoxvirusAbatino | - | NC_055231.1 | 94.60% |
| Variola virus | - | NC_001611.1 | 93.00% |
| Buffalopox virus | Karachi 2005 | MG599038.1 | 90.30% |
| Vaccinia virus | Dryvax | JN654982.1 | 90.30% |

**Figure legends**

**Figure S1. Confirmation of the newly developed conventional PCR for MPXV detection.** M, 100 bp marker. Lane 1-3 represent the products of ATI-plasmid, influenza virus A and DW.

**Figure S2. Temperature optimization for the MPX-LAMP assay**. The LAMP reactions for detecting MPXV at different temperatures were monitored by real-time turbiditor. A turbidity of >0.1 indicated positive amplification of LAMP. Eight kinetic graphs (A–H) were obtained at distinct temperatures ranging from 60 to 67 °C with 5×10^2^copies of ATI-plasmid as template. The graphs showed that 63°C was the optimal temperature for LAMP reaction.

**Figure S3. Sensitivity of the conventional PCR method for detection of MPXV.** The sensitivity of the conventional PCR for detection of MPXV was assessed by using plasmid templates (**A**) and pseudotyped virus templates (**B**) simultaneously. 1-8 in A represented different concentrations of ATI-plasmid ranging from 5×10^5^ to 5×10^-1^ copies per microliter per reaction; Tube/ Biosensor 8 represented the blank control (DW). 1-5 in B represented different concentrations of pseudotyped virus ranging from 1.25×10^3^ to 1.25×10^-2^ copies per microliter per reaction; 6 represented the blank control (DW). M, 100 bp marker.

**Figure S4. Time optimization for the MPX-LAMP-LFB assay.**

Two monitoring formats, including visual detection reagent (top row) and LFB (bottom row), were applied for LAMP product detection. Tubes/ Biosensors 1-7 represented different concentrations of ATI-plasmid ranging from 5×10^5^ to 5×10^-1^ copies per microliter per reaction; Tube/ Biosensor 8 represented the blank control (DW). TL, test line; CL, control line.

**Figure S5. Conventional PCR results of the non-MPXV infection clinical specimens.** 1-2 represented the results of ATI-plasmid and pseudotyped virus (positive control), 3-63 represented the results of the 61 NPSs from non-MPXV infection patients; and 64 represented the blank control (DW).

**Figure S6. Application of MPX-LAMP-LFB assay in clinical specimens.**

Biosensors 1-2 represented the LAMP reaction results of ATI-plasmid and pseudotyped virus, and Biosensors 3-63 represented the LAMP reaction results of the 61 NPSs from non-MPXV infection patients; and Biosensor 64 represented the blank control (DW). TL, test line; CL, control line

**Figure S7. Alignment of primers with other*Orthopoxvirus* members.** The primers for each of MPX-AMP-LFB assays were aligned with the targeted sequence of DNA within several orthopoxviral species. Virus strains:Monkeypox virus MPXV-USA2003_099_Gambian_Rat;Rabbitpox virus;Camelpox virus Negev2016; Horsepox virus MNR;Cowpox virusHumBer07/1;Taterapox virus;OrthopoxvirusAbatino;Variola virus; Buffalopox virusKarachi 2005;Vaccinia virusDryvax.
