# Supplementary figures and images for: Loop-mediated isothermal amplification combined with lateral flow biosensor for rapid and sensitive detection of monkeypox virus
Source: Front Public Health. 2023 Mar 24;11:1132896. doi: 10.3389/fpubh.2023.1132896 (PMC10080115; doi:10.3389/fpubh.2023.1132896)

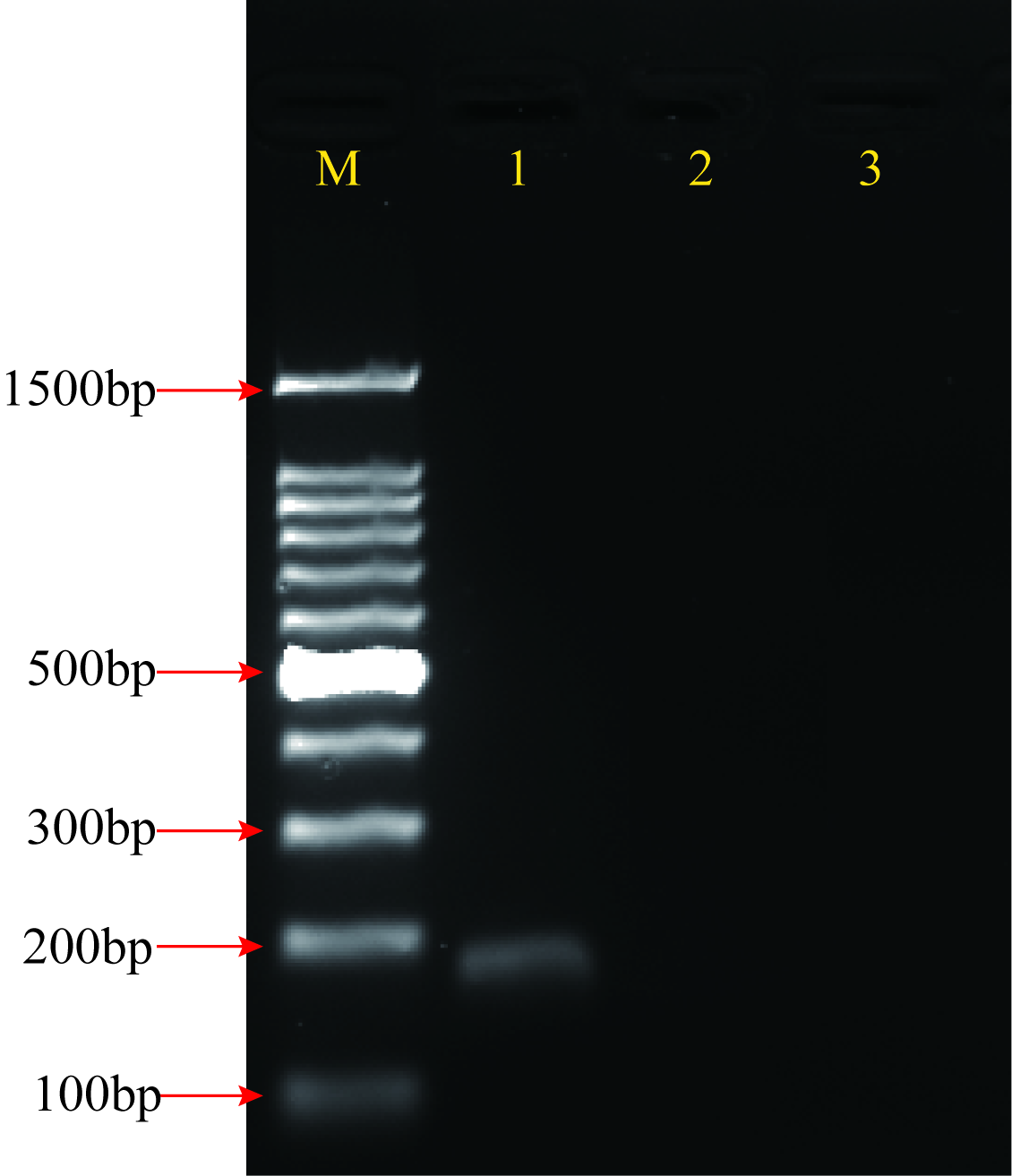

Supplement: Supplementary file 2 [file Image_1.TIF]

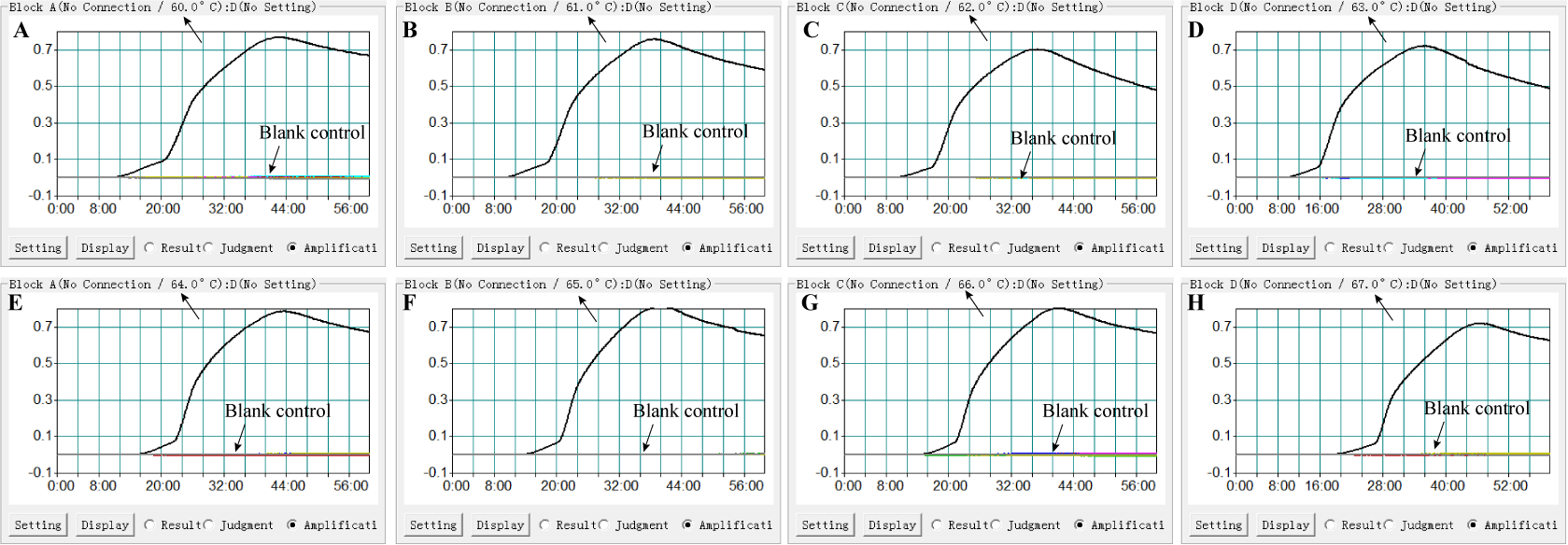

Supplement: Supplementary file 3 [file Image_2.TIF]

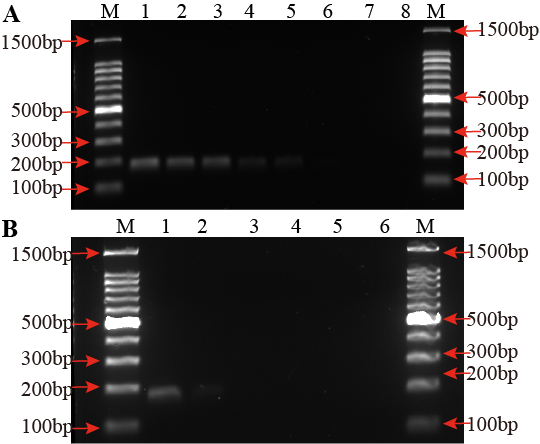

Supplement: Supplementary file 4 [file Image_3.TIF]

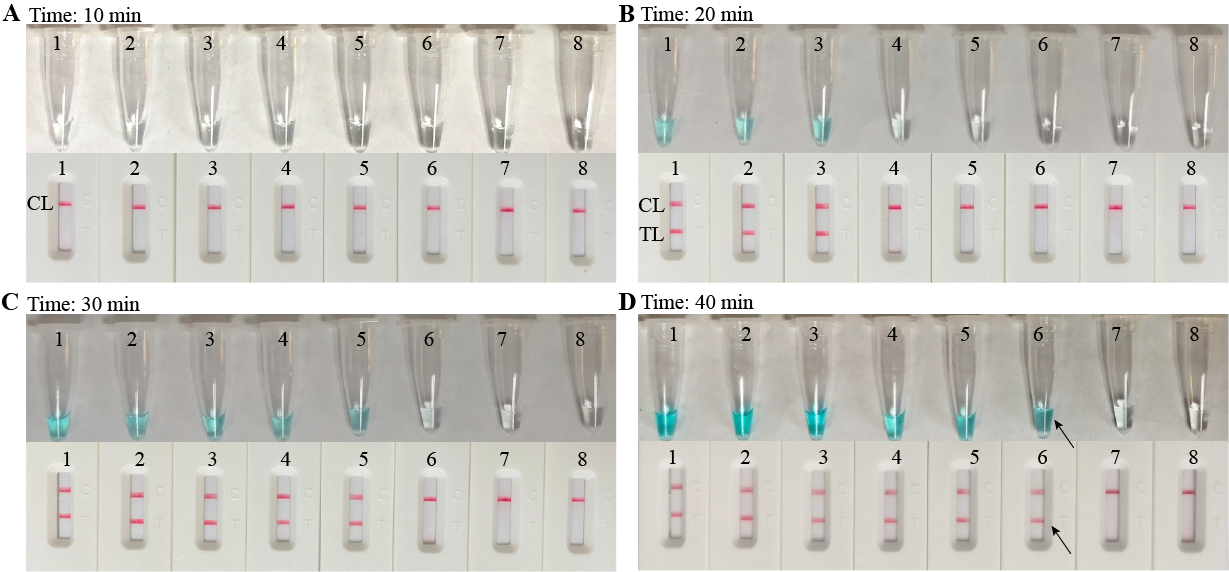

Supplement: Supplementary file 5 [file Image_4.TIF]

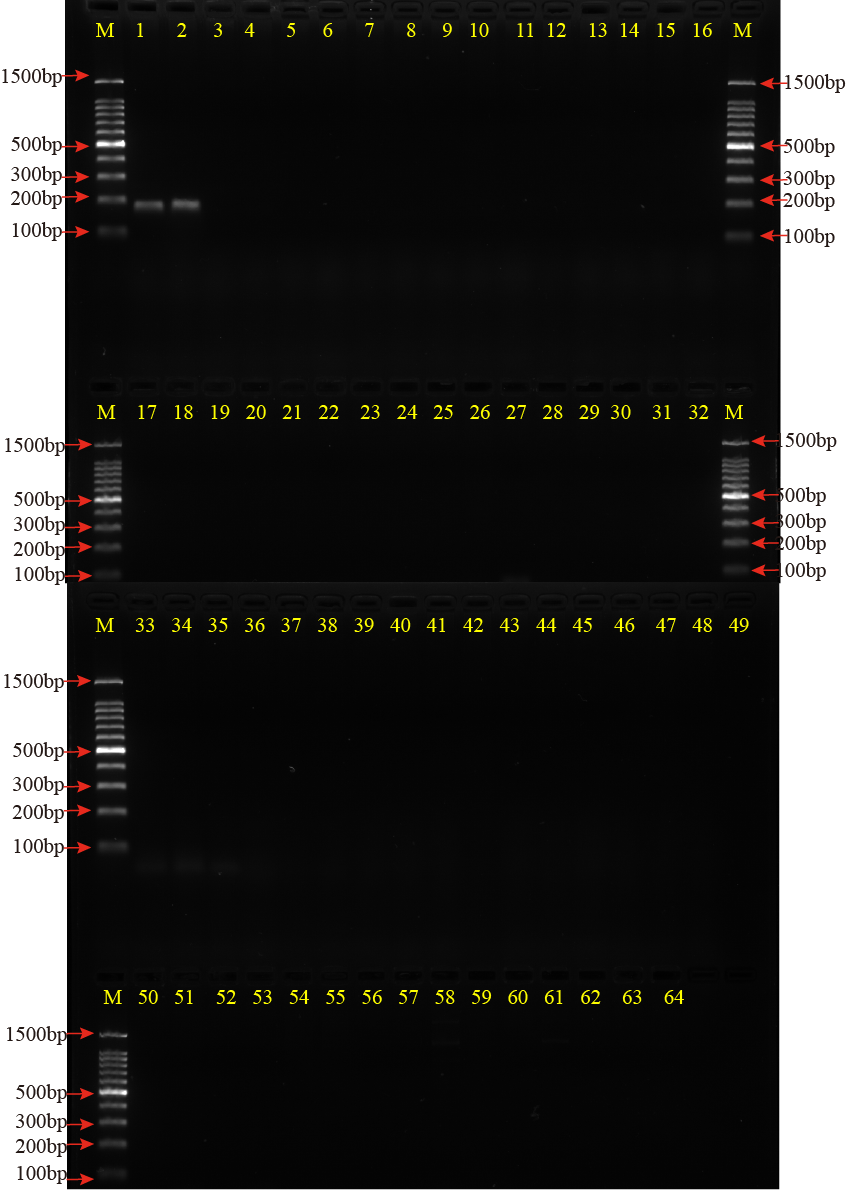

Supplement: Supplementary file 6 [file Image_5.TIF]

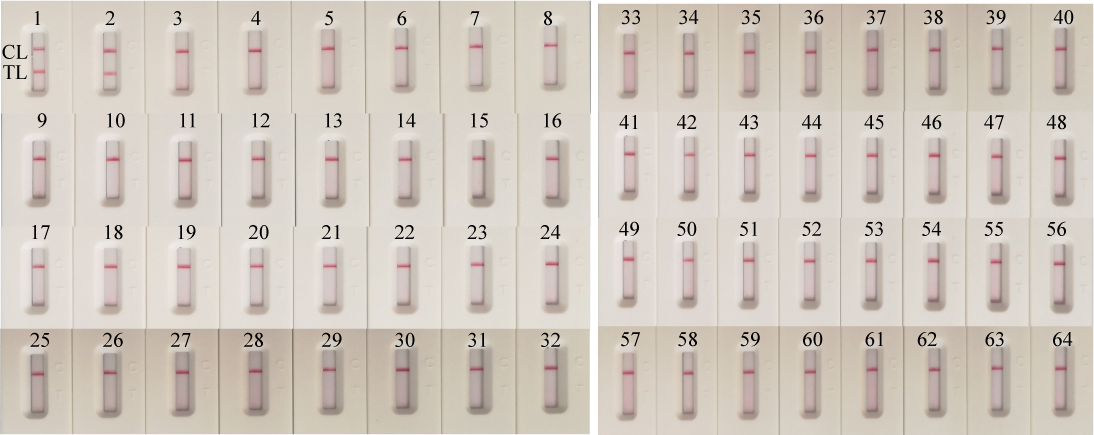

Supplement: Supplementary file 7 [file Image_6.TIF]

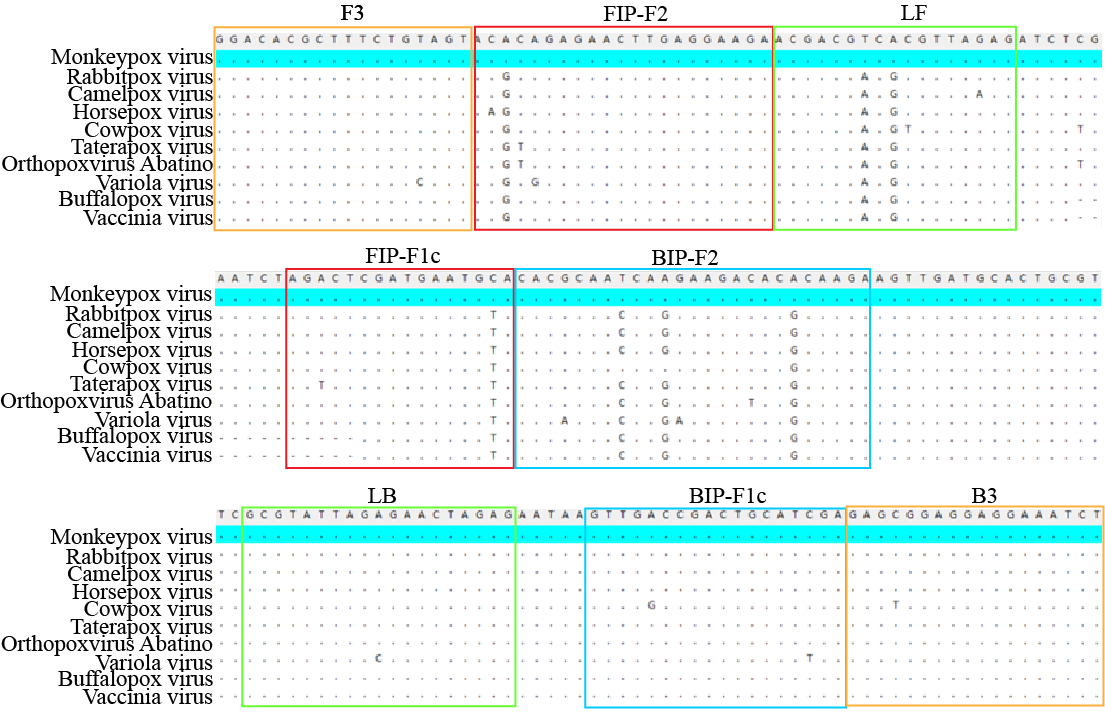

Supplement: Supplementary file 8 [file Image_7.TIF]
